# Synthesis of a Novel Disperse Reactive Dye Involving a Versatile Bridge Group for the Sustainable Coloration of Natural Fibers in Supercritical Carbon Dioxide

**DOI:** 10.1002/advs.201801368

**Published:** 2018-11-08

**Authors:** Yue Fan, Yan‐Qin Zhang, Kai Yan, Jia‐Jie Long

**Affiliations:** ^1^ College of Textile and Clothing Engineering Soochow University Suzhou 215123 China

**Keywords:** characterization, disperse reactive dyes, dyeing properties, supercritical carbon dioxide, synthesis

## Abstract

Disperse reactive dyes with appropriate chemical structure are key for the coloration of natural fibers in the water‐free environmentally friendly medium of supercritical carbon dioxide with various advantages. The objective of this work is to design and synthesize a novel anthraquinonoid disperse reactive dye involving a versatile bridge group to improve the coloration properties of the dye in supercritical carbon dioxide. Cross‐coupling condensation based on an Ullmann reaction between *N*‐phenylethylenediamine and 1‐chloroanthraquinone in a ligand‐free system is investigated by optimizing the synthesis parameters. Notable influences are observed from the dosages of *N*,*N*‐dimethyl formamide and potassium hydroxide, as well as the system temperature and reaction duration, on the isolated yield of the dye precursor. An optimized process is also recommended for synthesizing the designed novel dye. Then, the chemical structure, color characteristics, and coloration properties of the obtained dye are further investigated and successfully characterized by utilizing Fourier‐transform infrared analysis, ^1^H and ^13^C nuclear magnetic resonance spectroscopy, UV–vis absorption spectroscopy, elemental analysis, and liquid chromatography‐mass spectrometry. Additionally, practical coloration experiments are performed with cotton, silk, and wool in a supercritical carbon dioxide medium.

## 1 Introduction

In recent decades, systems involving dense gases under high pressures and temperatures above their individual critical points, namely, in the supercritical state, have demonstrated attractive properties for dissolving various organic substances.^1^ By taking advantage of the powerful dissolving behaviors of supercritical systems, Schollmeyer and co‐workers^2^ first evaluated such systems as dissolving and transport media for hydrophobic dyes to determine the potential of these systems, especially supercritical carbon dioxide, for textile dyeing. Moreover, to date, supercritical carbon dioxide can be readily employed as a dyeing medium in practice to completely avoid water consumption and wastewater discharge, which truly embodies the concept of green chemistry and sustainable alternatives with numerous advantages over conventional aqueous coloration methodologies.^3^, ^4^, ^5^, ^6^, ^7^, ^8^

However, to date, there is still a challenge in the coloration of natural fibers by utilizing supercritical carbon dioxide due to the unique properties of the supercritical fluid and the highly hydrophilic characteristics of the natural substrate.^3^, ^5^, ^6^, ^9^ Fortunately, as demonstrated from our previous work as well as other researchers' results,^3^, ^5^, ^6^, ^7^, ^8^, ^9^, ^10^ the application of a special kind of dye, namely, disperse reactive dyes, can meet most of the demands of both the supercritical medium and natural substrates during coloration via supercritical carbon dioxide. This method has attracted increasing attention in recent years not only due to its environmentally friendly advantages but also due to its facile and convenient implementation in practice.^9^, ^11^ Therefore, the appropriate design and synthesis of the chemical and conformation structures has become very important for developing a suitable and powerful disperse reactive dye for the coloration of different natural fibers in supercritical carbon dioxide fluid. Moreover, the supply of this kind of special dye is also very limited and not often found in the commercial market, although the demand from customers is quickly increasing with the rapid development of supercritical coloration technology.^2^, ^10^, ^11^, ^12^, ^13^

Theoretically, during the design and synthesis of the chemical and conformation structures of an individual disperse reactive dye, the bridge group or linking group between the chromophoric matrix and reactive group(s) also plays a crucial role in the coloration properties of dye uptake, adsorption, penetration, reactivity, fixation, and stability during application.^14^ Consequently, the appropriate selection of a bridge group is beneficial for improving the overall chemical and conformation structures and thus enhancing the application properties of a synthesized dye. Generally, imine (—NH—) and various substituted imine (—N(R)—) groups are usually employed as bridge groups in conventional reactive and/or disperse reactive dye structures. However, those conventional bridge groups, especially substituted imine groups with large bulky chains, usually create problems arising from steric hindrance during dye penetration in the networks of the fiber macrochains. These bridge groups frequently result in poor coloration results, such as low build‐up, uptake, and fixation. Furthermore, these effects can be amplified for disperse reactive dyes on hydrophilic fiber substrates in supercritical carbon dioxide. Consequently, it is important to properly design appropriate bridge groups in the chemical structures of disperse reactive dyes to eliminate and/or decrease steric hindrance in the penetration of dye molecules in fiber networks for improving the solubility of the dye in supercritical medium, etc., to achieve good coloration properties and results.

Moreover, to conveniently bond a bridge group onto a chromophoric matrix, the well‐known cross‐coupling condensation of the Ullmann reaction, which was derived by Fritz Ullmann in the early twentieth century,^15^ is usually employed. In particular, this approach is convenient for introducing C—C bonds into aromatic structures by utilizing the classical Ullmann‐type reaction via a reductive symmetrical coupling of aryl halides with copper metal as a catalyst.^16^ Furthermore, the conventional Ullmann‐type reaction has also been modified and much improved in recent decades for the formation of various carbon‐involved bonds, such as C—N, C—O, and C—S bonds,^17^, ^18^, ^19^, ^20^, ^21^, ^22^, ^23^, ^24^ especially for synthesizing various dyes and their intermediates in ligand‐free systems with numerous advantages.^9^, ^10^

In this work, a new disperse reactive dye with an anthraquinonoid chromophoric matrix and dichloro‐s‐triazine reactive group linked by a versatile bridge group of N‐phenylethylenediamine was designed and synthesized based on a copper‐catalyzed Ullmann reaction. The parameters of the cross‐coupling reaction, such as the dosages of the solvent (N,N‐dimethyl formamide (DMF)), acid‐bonding agent (potassium hydroxide (KOH)), catalyst (copper, Cu), the molar ratio of the reactants, and the reaction temperature and duration, were investigated and optimized. Furthermore, Fourier‐transform infrared (FT‐IR) analysis, ^1^H and ^13^C nuclear magnetic resonance (NMR) spectroscopy, liquid chromatography‐mass spectrometry (LC‐MS), and elemental analysis were also employed to confirm and validate the chemical structure. The color characteristics and dyeing properties of the obtained dye were further investigated and characterized by utilizing UV–vis absorption spectroscopy and practical coloration experiments with cotton, silk, and wool in supercritical carbon dioxide.

## 2 Results and Discussion

### 2.1 Effects of Synthesizing Parameters on the Isolated Yield of the Designed Dye Precursor

#### 2.1.1 The Dosage of Solvent

**Scheme 1:**
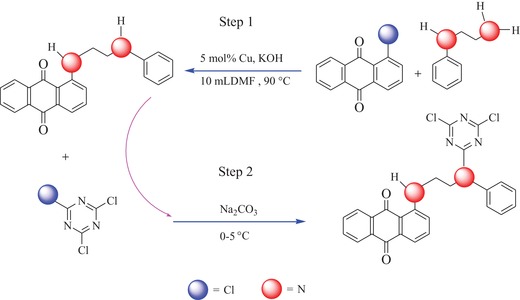
Synthetic route for the anthraquinone‐type disperse reactive dye.

The effect of the dosage of solvent (DMF) on the synthesis of the designed disperse reactive dye precursor was investigated on the basis of the Ullmann reaction by employing 2.00 mmol (0.485 g) of 1‐chloroanthraquinone and 2.00 mmol (260.0 µL) of N‐phenylethylenediamine as reactants, 0.20 mmol (0.013 g) metallic copper (Cu(0)) as a catalyst, 2.00 mmol (0.112 g) of KOH as an acid‐binding agent, and various volume dosages of DMF as the solvent in a ligand‐free system according to Step 1 in **Scheme** **1**. The reactions were carried out by stirring the mixtures for 10.0 h at 100.0 °C under a dry nitrogen atmosphere. The obtained results are summarized in **Table** **1**(i).

**Table 1:** The isolated yields of the dye precursor obtained under different conditions

Parameter		Isolated yield [%]
(i) Solvent dosage of DMF [mL]a)	5.0	49.64
	10.0	50.22
	15.0	41.46
	20.0	32.99
	25.0	26.28
(ii) Molar ratio of reactants (*n*_1_:*n*_2_)b),c)	1:10	50.22
	1:1.5	51.09
	1:20	51.85
	1:2.5	52.55
	1:30	53.43
(iii) Dosage of KOH [mmol]d)	1.0	43.36
	2.0	50.22
	3.0	51.30
	4.0	52.70
	5.0	51.39
(iv) Dosage of copper [mmol]e)	0	54.31
	0.1	60.15
	0.2	52.70
	0.3	52.41
	0.4	53.58
(v) Temperature [°C]f)	80	31.53
	90	60.88
	100	60.15
	110	56.20
	120	55.33
(vi) Time [h]g)	4.0	42.63
	6.0	48.47
	8.0	52.41
	10.0	60.88
	12.0	59.27

^a)^ Conditions: 2.0 mmol of 1‐chloroanthraquinone, N‐phenylethylenediamine, and KOH, in the presence of 0.2 mmol Cu at 100 °C under stirring for 10.0 h

^b)^ (*n*_1_:*n*_2_): refers to the molar ratio of 1‐chloroanthraquinone (*n*_1_) to N‐phenylethylenediamine (*n*_2_)

^c)^ Conditions: the mole ratios of 1‐chloroanthraquinone (*n*_1,_ 2.0 mmol) to N‐phenylethylenediamine (*n*_2_) were 1:1, 1:1.5, 1:2, 1:2.5, and 1:3 in the presence of 0.2 mmol Cu and 10.0 mL DMF at 100 °C under stirring for 10.0 h

^d)^ Conditions: 2.0 mmol of 1‐chloroanthraquinone and 2.0 mmol of N‐phenylethylenediamine in the presence of 0.2 mmol Cu and 10.0 mL DMF at 100 °C under stirring for 10.0 h

^e)^ Conditions: 2.0 mmol of 1‐chloroanthraquinone and 2.0 mmol of N‐phenylethylenediamine in the presence of 4.0 mmol KOH and 10.0 mL DMF at 100 °C under stirring for 10.0 h

^f)^ Conditions: 2.0 mmol of 1‐chloroanthraquinone, 2.0 mmol of N‐phenylethylenediamine, and 4.0 mmol KOH in the presence of 0.1 mmol Cu and 10.0 mL DMF under stirring for 10.0 h

^g)^ Conditions: 2.0 mmol of 1‐chloroanthraquinone, 2.0 mmol of N‐phenylethylenediamine, and 4.0 mmol of KOH in the presence of 0.1 mmol Cu and 10.0 mL DMF under stirring at 90 °C.

Table 1(i) shows that a notable decreasing tendency in the isolated yield of the achieved dye precursor was observed as the solvent dosage of DMF increased from 10.0 to 25.0 mL in the ligand‐free copper‐catalyzed Ullmann reaction system, accompanied by a maximum yield of 50.22% with a DMF volume of 10.0 mL. These results clearly indicate that the dosage of the solvent (DMF) plays an important role in the production ratio of the designed dye precursor, probably because an appropriate amount of the solvent could provide adequate concentrations of the reactants in a homogeneous state in the reaction system, which led to an accelerated reaction rate and an improved total conversion of the target product. Generally, if the dosage of the solvent (DMF) is too low, the solid reactant 1‐chloroanthraquinone and the acid binding agent KOH are partly dissolved in the solvent, which readily decreases the total conversion of the product in a fixed duration. Moreover, challenges in operation, such as the inhomogeneous stirring of the reaction system, are also encountered. However, an excess dosage of solvent readily decreased the concentrations of the reactants, the catalyst and the base agent in the system, which resulted in a low reaction rate and a reduced isolated yield of the dye precursor. Therefore, a solvent (DMF) dosage of 5.0–10.0 mL was recommended for the following experiments.

#### 2.1.2 The Molar Ratio of Reactants

The effect of the utilized molar ratio of the aryl chloride reactant (1‐chloroanthraquinone (*n*_1_, 2 mmol)) to the nucleophile of N‐phenylethylenediamine (*n*_2_) on the isolated yield of the dye precursor was also investigated by employing the copper‐mediated Ullmann cross‐coupling reaction in the presence of 0.2 mmol Cu catalyst and 10.0 mL DMF as a medium. The reaction was performed at 100 °C by stirring for 10.0 h under a dry nitrogen atmosphere. The achieved results are summarized in Table 1(ii).

Table 1(ii) shows that a slightly increasing tendency in the isolated yield of the designed dye precursor was observed with the increase in the molar ratio of the employed reactants (*n*_1_:*n*_2_) in the metallic copper‐catalyzed reaction system. An isolated yield of ≈50.22% was achieved with a reactant ratio (*n*_1_:*n*_2_) of 1:1, and a relatively high yield at 53.43% was observed as the reactant ratio (*n*_1_:*n*_2_) was increased to 1:3. As described from Le Châtelier's principle,^25^ the copper‐catalyzed Ullmann‐type coupling reaction equilibrium is readily shifted in the positive direction by improving the utilization of the nucleophile reactant of N‐phenylethylenediamine (*n*_2_), which benefits for the production of the designed dye precursor. Accordingly, an improvement in the dye precursor yield was observed by increasing the molar ratio of N‐phenylethylenediamine (*n*_2_) due to an improvement in the conversion of 1‐chloroanthraquinone. However, from the perspective of reaction economy, a molar ratio of 1‐chloroanthraquinone (*n*_1_) to N‐phenylethylenediamine (*n*_2_) of 1:1 is recommended for synthesizing the designed dye precursor.

#### 2.1.3 Dosage of the Base Agent

To improve the nucleophilic substitution reaction of N‐phenylethylenediamine during the copper‐mediated Ullmann‐type condensation reaction, a base agent of KOH was used as an acid binding agent with concentrations ranging from 1.0 to 5.0 mmol in the presence of 2.0 mmol of 1‐chloroanthraquinone, 2.0 mmol of N‐phenylethylenediamine, 0.2 mmol Cu as the catalyst and 10.0 mL DMF as the solvent. Then, the reaction was carried out at 100 °C by stirring for 10.0 h under a dry nitrogen atmosphere. The results are depicted in Table 1(iii).

As shown in Table 1(iii), a notable enhancement in the isolated yield for the dye precursor was obtained with dosages of KOH from 1.0 to 4.0 mmol, accompanied by a maximum value of 52.70% for the dye precursor yield. However, a further increase in the dosage of KOH to 5.0 mmol in the copper‐catalyzed Ullmann system did not produce a further enhancement in the isolated dye precursor yield. Theoretically, as depicted in the reaction route in Scheme 1, a base agent is necessary to improve the nucleophilicity of N‐phenylethylenediamine and act as an acid binding agent during the exchange or nucleophilic substitution of the reactant with the activated chloride atom on copper species. It is also helpful to shift the reaction equilibrium in the positive direction for the convenient formation of the organocopper intermediates in the Ullmann coupling system. Therefore, all of these factors lead to enhanced isolated yield with an appropriate increase in the dosage of the base agent. However, a further increased dosage of the base agent in the copper‐catalyzed Ullmann condensation system probably presented some negative effects on the catalyst species due to side reactions, such as reactions between hydroxyl (OH^−^) and various copper ions, especially the cuprous ions released from the different reductive elimination steps in the system, which finally resulted in the decrease in the isolated yield. Consequently, a dosage of 4.0 mmol KOH was recommended for dye precursor synthesis via the metallic copper‐catalyzed Ullmann reaction system in subsequent experiments.

#### 2.1.4 Dosage of Metallic Copper Catalyst

The utilization of copper catalyst species is essential to successfully and economically carry out all copper‐mediated Ullmann‐type cross‐coupling reactions. Therefore, the effect of the dosage of metallic copper catalyst on the isolated yield of the dye precursor was also explored with copper catalyst dosages ranging from 0.0 to 0.4 mmol and the presence of 2.0 mmol of 1‐chloroanthraquinone, 2.0 mmol of N‐phenylethylenediamine, 4.0 mmol of KOH, and 10.0 mL DMF as solvent. The reaction was carried out at 100 °C by stirring for 10.0 h under a dry nitrogen atmosphere. The achieved results are shown in Table 1(iv).

Table 1(iv) demonstrates that a notable enhancement in the isolated yield of over 60% for the dye precursor was observed as a catalytic amount of metallic copper catalyst at 0.1 mmol was added to the reaction system, although some decreases were also encountered with higher dosages of the metallic copper catalyst. In principle, as observed in copper‐mediated Ullmann reactions in the literature,^17^, ^26^ the crucial role of the copper catalyst is to activate the aryl halide compound of 1‐chloroanthraquinone by an oxidative addition reaction to form different temporary organocopper intermediates with the copper species. Consequently, appropriate addition of the copper catalyst is helpful to promote and facilitate the subsequent exchange and nucleophilic substitution with the nucleophile of N‐phenylethylenediamine, which readily enhances the isolated yield. However, excess dosages of the metallic copper catalyst in the reaction system could readily present undesired side reactions between the copper species and the reactants via undesired chelation, etc., as well as reactions with the base agent via the ionic reaction of hydroxyl (OH^−^) and different copper ions in the system. These effects resulted in the reduction of the isolated yield of the dye precursor in Table 1(iv). Therefore, a catalytic amount of the metallic copper catalyst at 0.1 mmol was recommended in the following experiments.

#### 2.1.5 Reaction Temperature and Duration

Reaction system temperature and duration are also important parameters for the production of the dye precursor in the Ullmann‐type reaction system. Accordingly, the effects of system reaction temperature and duration on the synthesis of the designed dye precursor were investigated by utilizing 2.0 mmol of 1‐chloroanthraquinone, 2.0 mmol of N‐phenylethylenediamine, 4.0 mmol KOH, 0.1 mmol of metallic Cu as catalyst, and 10.0 mL DMF as solvent in the reaction system. The results are summarized in Table 1(v,vi).

As shown in Table 1(v), a significant improvement in the isolated yield of the dye precursor was achieved upon increasing the reaction temperature from 80 to 90 °C, accompanied by a maximum isolated yield of 60.88%. However, a gradual decreasing tendency was also observed with a further increase in the system temperature up to 120 °C. Generally, an appropriate reaction temperature can readily promote the copper‐mediated Ullmann cross‐coupling reaction for C—N bond formation with an enhanced conversion rate.^17^, ^26^ In particular, it is also helpful to initiate the oxidative addition of the aryl halide compound of 1‐chloroanthraquinone to copper species catalysts to form the organocopper intermediates. Therefore, all these factors resulted in the significant improvement of the isolated yield. Additionally, the gradual decreasing tendency is possibly due to more serious side reactions, as described above, which occurred among the reactants, catalyst species, and base agent at an excessively high reaction temperature. Consequently, a reaction temperature at 90 °C was recommended for synthesizing the dye precursor in the employed copper‐mediated Ullmann condensation system.

Furthermore, Table 1(vi) reveals that a notable enhancement in the isolated yield of the dye precursor was observed with reaction durations from 4.0 to 10.0 h, and no further improvement was achieved as the duration was extended to 12.0 h. Theoretically, in a typical ligand‐free copper‐mediated Ullmann cross‐coupling reaction, a relatively long duration is usually necessary to achieve a satisfactory conversion rate, especially when using a metallic copper species as the catalyst in a heterogeneous reaction system, in which a relatively low mass transfer efficiency is usually encountered among the interfaces of solid–liquid phases. Consequently, a reasonably extended duration led to the enhancement of the isolated yield in Table 1(vi), and a reaction duration of 10.0 h was recommended for synthesizing the dye precursor in the metallic copper‐mediated Ullmann condensation system.

### 2.2 Characterization of the Disperse Reactive Dye with a Versatile Bridge Group

The structures of the synthesized dye precursor and its final product were characterized by Fourier‐transform infrared spectroscopy, nuclear magnetic resonance, elemental analysis, and LC‐MS. Relevant results were obtained and are shown as follows.

#### 2.2.1 FT‐IR Analysis of the Dye Precursor and Its Final Product

**Figure 1:**
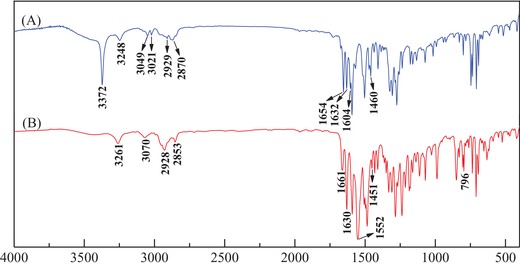
FT‐IR spectra of A) the precursor and B) the anthraquinone‐type disperse reactive dye.

The synthesized anthraquinonoid dye precursor and the final product with versatile bridge groups were analyzed and confirmed by FT‐IR spectra. The recorded spectra are shown in **Figure** **1**A,B. The FT‐IR spectrum for the anthraquinonoid dye precursor in Figure 1A shows that a sharp and clear peak was observed at 3372 cm^−1^, which was attributed to the symmetrical stretching vibration from the secondary amino group (—NH—) at the α‐position of the versatile bridge group of N‐phenylethylenediamine. Furthermore, a normal strong characteristic peak at ≈3474 cm^−1^, which corresponds to the asymmetric N—H stretching vibration of the amino group (—NH_2_) in N‐phenylethylenediamine, was not observed in Figure 1A. By contrast, a small characteristic peak at 3248 cm^−1^ was recorded, which was associated with the symmetrical stretching vibration of the secondary amino group (—NH—) at the β‐position of N‐phenylethylenediamine. This clearly reveals that the metallic copper‐catalyzed Ullmann cross‐coupling reaction occurred between the chloro group (—Cl) of 1‐chloroanthraquinone and the amino group (—NH_2_) on N‐phenylethylenediamine to produce the dye precursor, as depicted in Step 1 of Scheme 1. The shift in the peak for the stretching vibration of the β‐position secondary amino group (—NH—) produced from the cross‐coupled N‐phenylethylenediamine to a low wavenumber at 3248 cm^−1^ was probably due to the formation of an intramolecular hydrogen bond with the carbonyl in the dye precursor structure. Additionally, N—H bending vibrations from the two kinds of secondary amino groups were also observed at 1632 and 1604 cm^−1^, which further confirmed the formation of the C—N bond between the two reactants via the copper‐catalyzed Ullmann cross‐coupling reaction. Furthermore, stretching vibrations from the =CH bonds in the aromatic rings of the synthesized dye precursor were observed at 3409 and 3021 cm^−1^, as shown in Figure 1A; the asymmetric and symmetric stretching vibrations and bending vibrations of C—H bonds from the methylene groups (—CH_2_—) in the dye precursor at 2929, 2870, and 1460 cm^−1^, respectively, were also observed. A characteristic stretching vibration of the carbonyl bond (C=O) was also observed at 1654 cm^−1^. These results clearly prove that the designed dye precursor was successfully synthesized by utilizing the copper‐mediated Ullmann‐coupling reaction between the employed reactants.

Figure 1B demonstrates that the sharp and strong peak at 3372 cm^−1^ belonging to the symmetrical stretching vibration of the secondary amino group (—NH—) at the α‐position of the versatile bridge group of N‐phenylethylenediamine disappeared, as did its bending vibration peak at 1604 cm^−1^ after a nucleophilic substitution reaction between the dye precursor and cyanuric chloride, as shown in Step 2 of Scheme 1. This evidently reveals that the hydrogen atom (—H) in the free secondary amino group (—NH—) at the α‐position of the versatile bridge group was substituted by the active chloro group (—Cl) from cyanuric chloride to produce the final product of the novel disperse reactive dye involving a dichloro‐S‐triazine reactive group. Moreover, a characteristic absorption peak at 1522 cm^−1^, which was attributed to the C=N stretching vibration from the s‐triazine ring, was also achieved. A C—Cl stretching vibration at the active site of dichloro‐S‐triazine at 796 cm^−1^ was also observed and is shown in Figure 1B. These results further prove that the designed anthraquinonoid disperse reactive dye involving a versatile bridge group and a reactive group(s) of substituted cyanuric chloride was successfully synthesized by employing the copper‐catalyzed Ullmann reaction and a subsequent nucleophilic substitution, as shown in Scheme 1.

#### 2.2.2 ^1^H NMR and ^13^C NMR Spectral Analysis of the Dye Precursor and Its Final Product

**Figure 2:**
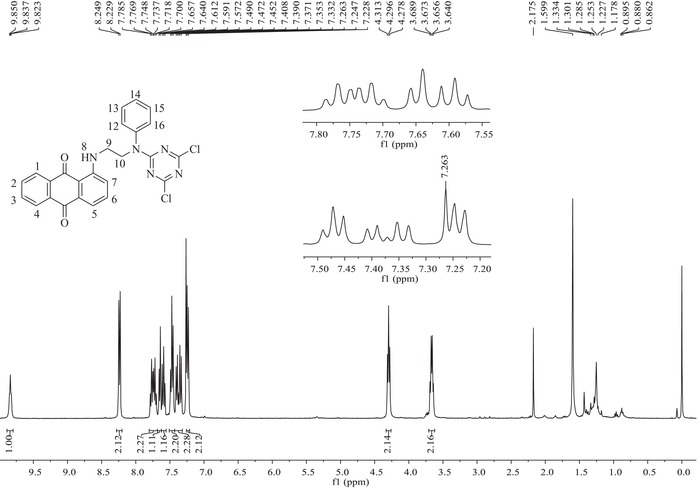
^1^H NMR spectra of the anthraquinone disperse reactive dye.

**Figure 3:**
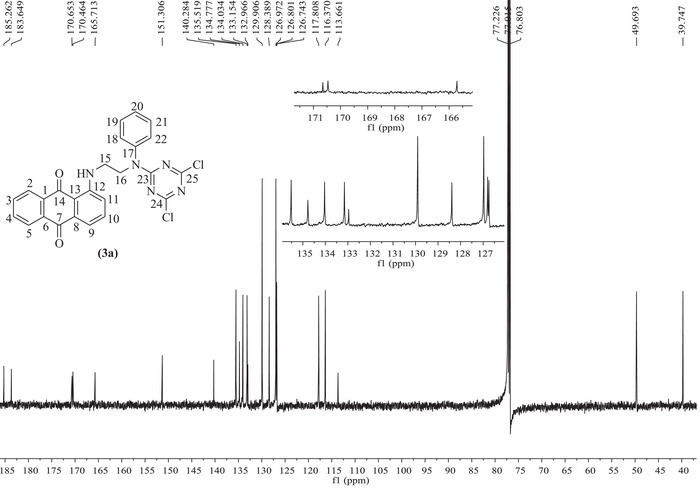
^13^C NMR spectra of the anthraquinone‐type disperse reactive dye measured in DMSO‐d_6_.

The synthesized dye precursor and its final product, the novel disperse reactive dye, were further analyzed and characterized by employing ^1^H NMR and ^13^C NMR spectral detection in CDCl_3_ as solvent after column chromatography purification. The achieved results are shown in **Figures** **2** and **3** as well as in Figure S1 in the Supporting Information.

Figure 2 is the ^1^H NMR spectrum of the synthesized disperse reactive dye; these data were used to characterize and confirm the protons connected to the carbon atoms in the dye molecular structure. As shown in Figure 2 and the summarized characteristic data in the subsection “The Obtained Characteristic Data for the Chemical Structure and Properties of the Dye Precursor and Its Final Product” of the Experimental Section, the corresponding and quantitative protons from the anthraquinone backbone ring, such as H‐(1,4; 2,3; 5; 6; and 7), were all detected with chemical shifts (δ) in a range from 8.24 to 7.32 ppm accompanied by individual and characteristic line splitting, respectively. Moreover, the corresponding protons of H‐(14; 13, 15; and 12,16) from the benzene ring of the substituted versatile bridge were also observed at chemical shifts (δ) of 7.43 to 7.32, 7.47, and 7.24 ppm, respectively, along with their characteristic line splitting. Additionally, a triplet at 4.30 ppm for the two protons of H‐10 and a double doublet at 3.66 ppm for the H‐9 protons from the aliphatic chain of the bridge group were also successfully detected. Importantly, a characteristic and evident singlet with a chemical shift (δ) at 9.84 ppm attributed to the imino (—NH—) proton of H‐8 at the α‐position of the anthraquinone ring was detected. These results further prove that copper‐mediated Ullmann cross‐coupling condensation occurred during synthesis of the dye precursor and demonstrate the successful achievement of the dye precursor. In particular, the singlet with a chemical shift (δ) at 3.98 ppm for the imino (—NH—) proton of H‐11′ (shown in Figure S1 of the Supporting Information and the summarized data for the dye precursor) was absent from the final product of the disperse reactive dye in Figure 2. This clearly reveals that the reactive group of cyanuric chloride was successfully bonded onto the dye precursor via the versatile bridge group to achieve the final product by employing the condensation reaction between the reactants according to the proposed reaction route of step 2 in Scheme 1.

Furthermore, Figure 3 shows the ^13^C NMR spectra for the final product of the synthesized disperse reactive dye. All the C‐(1‐14) carbon atoms from the anthraquinone backbone ring were detected, with chemical shifts (δ) ranging from 185.26 to 113.66 ppm. Moreover, the C‐(17‐22) carbon atoms from the benzene ring of the versatile bridge were also observed with chemical shifts (δ) from 151.31 to 116.37 ppm. The C‐15 and C‐16 carbon atoms from the ethyl chain of the bridge group were also detected at chemical shifts (δ) of 39.75 and 49.69 ppm, respectively. Additionally, the characteristic carbons of C‐(23‐25) from the S‐triazine ring were detected with chemical shifts (δ) at 170.65 and 170.46 ppm. Therefore, these results further demonstrate that the expected carbon backbone structure was successfully achieved for the disperse reactive dye, according to the designed chemical structure and synthesis route in Scheme 1.

#### 2.2.3 UV–Vis Absorption Spectral Analysis of the Dye Precursor and Its Final Product

**Figure 4:**
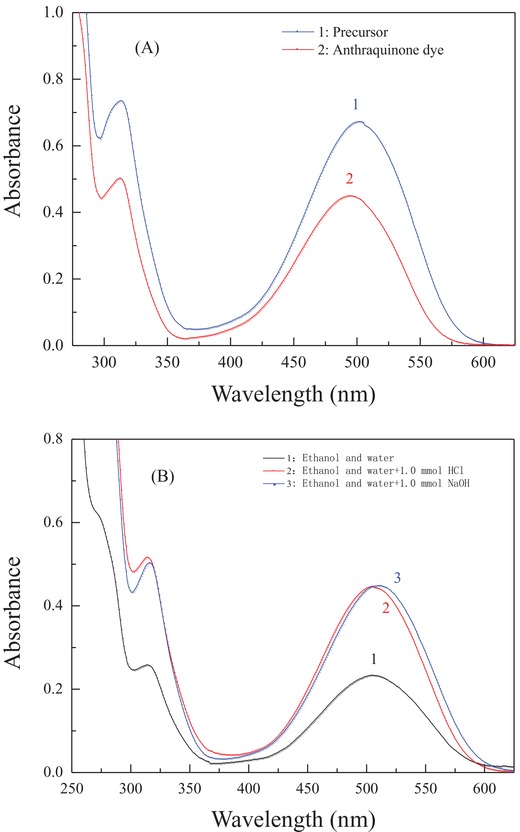
UV–vis absorption spectra of the anthraquinone‐type disperse reactive dye in different media: A) dichloromethane, B) diluted acidic and alkaline solutions.

UV–vis absorption spectral analysis was also performed to investigate the color characteristics of the synthesized disperse reactive dye and its precursor in the solvents of dimethylsulfoxide (DMSO), DMF, ethanol, dichloromethane, and *n*‐hexane with dye concentrations of 1.0 × 10^−4^–1.5 × 10^−4^ mol L^−1^. The obtained results are shown in **Figure** **4** and **Table** **2**.

**Table 2:** Influences of different solvents on the UV–vis absorption of the synthesized anthraquinone dye

Solvent	Dye concentration [ × 10^−5^, mol L^−1^]	Maximum absorption wavelength [λ_max,_ nm]	Molar absorption coefficient ε_max_ [ × 10^3^, L (mol cm)^−1^]
DMSO	13.5	504	6.80
DMF	11.2	501	5.59
Ethanol	11.6	496	5.75
Dichloromethane	14.9	495	7.36
N‐hexane	10.2	481	4.92

Figure 4A shows that the synthesized dye precursor and its disperse reactive dye both presented a characteristic single absorption peak with good shape and high intensity at a visible wavelength range of 375.0–600.0 nm in dichloromethane. Moreover, a maximum absorption wavelength (λ_max_) of the dye precursor at 502.0 nm was detected, and a slight shift in this maximum to 495.0 nm in the final disperse reactive dye product was also determined. Obviously, an inductive effect occurred to lead to the wavelength shift due to the bonding of the electron‐withdrawing reactive group of dichloro‐S‐triazine onto the dye precursor system, as well as the effect of increased steric hindrance from the bound reactive group on the coplanarity of the dye.

Table 2 shows the solvation effects of different solvents with various polarities on the chromophoric matrix and the color characteristics of the synthesized disperse reactive dye. Evident solvation effects from the solvents were observed on the conjugated system of the synthesized anthraquinone dye: a significant hypsochromic shift in the maximum absorption wavelength (λ_max_) from 504 to 481 nm was observed with decreasing polarity, from DMSO to the nonpolar solvent *n*‐hexane. These observations clearly indicate that the achieved disperse reactive dye involving an anthraquinone chromophoric matrix and the designed versatile bridge group tends to interact more closely with the polar solvents, which simultaneously affects the electronic cloud density of the dye conjugation system. Theoretically, strong polar solvents, such as DMSO or DMF, which have high values of the Kamlet–Taft parameters α, β, and π*,^27^ are able to interact with the functional groups from the dye solute, such as carbonyl (C=O) and imino (—NH—) groups, via hydrogen bonds by acting as H‐bond donors and/or acceptors. Additionally, other kinds of physical intermolecular interactions, such as the orientation force, the induction force, and the dispersion force, also readily intensify the interactions between polar solvents and the dye solute.^27^ Consequently, the electronic cloud density of the dye conjugation system was affected by polar solvents during the interactions, especially due to the formation of hydrogen bonds, which resulted in the variations in the chromophoric properties and the color hue of the synthesized dye in Table 2. Thus, different color hues from orange to red were observed as the dye solute dissolved in different solvents with different polarities. Additionally, relatively high molar absorption coefficients of 4.92 × 10^3^–7.36 × 10^3^ (ε_max_, L (mol cm)^−1^) were obtained whether in the highly polar solvent DMSO or in the hydrophobic solvent *n*‐hexane. These results demonstrate that the designed and successfully synthesized anthraquinonoid disperse reactive dye in this work has satisfactory color characteristics and sound chromophoric properties in different media.

Figure 4B further reveals the acid–base effects from the utilized media on the chromophoric properties and color hue of the synthesized disperse reactive dye. As shown in the spectrophotometric curves, good characteristic absorption peaks were detected with wavelengths ranging from ≈400.0 to 600.0 nm for neutral, acidic, and basic media. Moreover, a similar maximum absorption wavelength (505.0 nm) of the dye solute was observed in the neutral medium of ethanol mixed with water and the acidic medium of ethanol mixed with water and 1.0 mmol HCl. A slightly bathochromic shift to 510.0 nm in the maximum absorption wavelength of the dye solute was also achieved in the basic media of ethanol mixed with water and 1.0 mmol NaOH. Accordingly, all these results clearly indicate that the achieved disperse reactive dye is more stable in neutral and acidic media than in basic media in terms of color characteristics, etc.

In addition, the results from elemental and LC‐MS analysis were also determined, as shown in the subsection “The Obtained Characteristic Data for the Chemical Structure and Properties of the Dye Precursor and Its Final Product” of the Experimental Section and in Figure S2 (Supporting Information). These results further demonstrate that the content of C, H, and N in the dye molecules, as well as the relative molecular weight, agreed well with the values from theoretical calculations. The quasimolecular ion peaks and isotopic peaks of the target dye molecules were also successfully observed as the products were separated and detected by mass spectrometry using LC‐MS analysis.

### 2.3 Possible Reaction Mechanism for Synthesizing the Dye Precursor with a Copper Catalyst

All the above achieved results, including the different isolated yields of the target product in Table 1, the successful characterization and confirmation of the designed dye precursor, and its final product, clearly demonstrate that a typical Ullmann‐type cross‐coupling reaction occurred and was successfully carried out between an aryl halide (1‐chloroanthraquinone) and a nucleophile (N‐phenylethylenediamine) via the addition of a catalytic amount of metallic copper (Cu^(0)^) as a catalyst in the reaction system under a mild condition of 100.0 °C. To date, there are more than four kinds of proposed fundamental pathways for elucidating the mechanism of Ullmann‐type reactions; these pathways include the typical oxidative addition/reductive elimination mechanism, the aryl radical or single electron transfer mechanism, the σ‐bond metathesis mechanism, and π‐complexation mechanism.^17^, ^26^ Among all the proposed mechanistic pathways, the typical oxidative addition/reductive elimination mechanism is favored in the interpretation of copper‐mediated Ullmann condensation reactions. Moreover, various copper sources are effective, and cuprous ions (Cu^(1)^) are generally accepted as the primary and main reactive species for catalysis, although all the supposed mechanisms are still controversial and debated.^17^

**Scheme 2:**
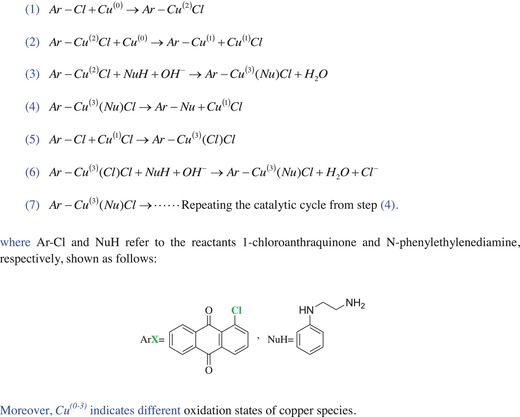
Proposed oxidative addition/reductive elimination mechanism for synthesizing the precursor based on Ullmann reaction with a copper catalyst.

In this work, an oxidative addition/reduction elimination mechanism based on the Ullmann reaction involving metallic copper as a catalyst is also proposed, as shown in **Scheme** **2**, for synthesizing the designed disperse reactive dye precursor. Accordingly, among the proposed different possible reaction procedures shown in Scheme 2(1–7), the initial oxidative addition of the aryl halide (1‐chloroanthraquinone) to the metallic copper catalyst could form an organocopper intermediate of Ar–Cu^(2)^Cl with a copper^(2)^ oxidation state,^17^, ^26^ as shown in step (1) of Scheme 2. Moreover, the formed organocopper intermediate (Ar–Cu^(2)^Cl) could react with the metallic copper catalyst further by an oxidation–reduction reaction, as shown in step (2), to produce the active and effective catalyst of cuprous chloride (Cu^(1)^Cl) and the organocopper intermediate species Ar–Cu^(1)^.^17^, ^26^ Furthermore, as depicted in step (3), the active species of the organocopper intermediate (Ar–Cu^(2)^Cl) could also react with the nucleophile N‐phenylethylenediamine (NuH) in the presence of the base via the oxidative addition of the nucleophile (NuH) onto the copper to form a temporary organocopper intermediate (Ar–Cu^(3)^(Nu)Cl) with an oxidized copper species state.^17^ The achieved temporary organocopper intermediate Ar–Cu^(3)^(Nu)Cl is usually not stable, and a reductive elimination reaction occurs readily, producing the designed anthraquinonoid dye precursor and releasing the effective catalyst Cu^(1)^Cl in step (4).^17^, ^26^ Therefore, as shown in step (5) of Scheme 2, the release of the active Cu^(1)^Cl catalyst from steps (2) and (4) could further activate the aryl chloride reactant (1‐chloroanthraquinone (ArX)) by oxidative addition with the copper species and form a new temporary organocopper intermediate (Ar–Cu^(3)^(Cl)Cl).^17^, ^26^ Then, one of the activated chloride atoms could be exchanged by the nucleophile N‐phenylethylenediamine (NuH) to form the temporary organocopper intermediate Ar–Cu^(3)^(Nu)Cl in step (6). Finally, the obtained intermediate (Ar–Cu^(3)^(Nu)Cl) readily produces the expected cross‐coupling dye precursor in step (7) as well as in step (4), and the catalytic reaction cycle can also proceed from step (4) to step (7).

Additionally, the initial metallic copper catalytic reaction can occur continuously from step (1) to (4) to produce the effective cuprous chloride (Cu^(1)^Cl) catalyst, as well as the designed dye precursor. Consequently, all the possible paths and proposed mechanism of the oxidative addition/reductive elimination reactions successfully resulted in the achievement of the desired dye precursor in the utilized system.

### 2.4 Dyeing Properties of the Synthesized Disperse Reactive Dye on Cotton, Silk, and Wool

The designed and synthesized disperse reactive dye involving a versatile bridge group was also applied to dye cotton, silk, and wool fabrics in supercritical carbon dioxide. The dye uptake, color characteristics, leveling performance, and color fastness on the substrates were investigated.

#### 2.4.1 Dye Uptake, Color Characteristics, and Leveling Performance

**Figure 5:**
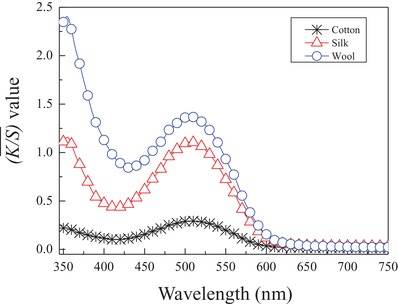
The color intensities A) of cotton (‐*‐), silk (‐△‐), and wool (…○…) substrates dyed with the synthesized anthraquinone disperse reactive dye at a dosage of 1.0% (o.m.f.) with 0.5% (v/v) acetone in supercritical carbon dioxide under the conditions of 20.0 MPa, 120 °C, and 90 min with a time ratio of fluid circulation to static treatment of 0.10, as well as B) the dye fixation efficiencies.

The results of the dye uptake and its color characteristics on cotton, silk, and wool fabrics in supercritical carbon dioxide are shown in **Figure** **5** and **Table** **3**. Figure 5 indicates that a single, well‐defined, and relatively sharp absorption peak ranging from 425.0 to 600.0 nm with a maximum characteristic absorption wavelength at 505.0 nm was obtained for each substrate. This peak reveals that a bright red shade was achieved on the cotton, silk, and wool fabrics by employing the synthesized disperse reactive dye. Moreover, the highest color intensity, with a $\bar{K/S}$ value (λ_max_) of 1.373, was observed for the wool substrate by utilizing the disperse reactive dye under the same dyeing conditions in supercritical carbon dioxide, followed by the silk and cotton substrates in order, as shown in Figure 5 and Table 3. The colorimetric data for the utilized substrates presented different colorimetric characteristics, such as the color lightness (L*), color‐opponent dimension values (a* and b*), chroma (C*), and hue (H*) in the CIE 1976 (L*, a*, b*) color space model, which are shown in Table 3. These results further reveal that a perfect and more brilliant color with a red hue and higher intensity is available on wool and silk than on cotton. Additionally, very low values (lower than 0.066) of the standard deviations (σ_K/S_) from the $\bar{K/S}$ values (λ_max_) were also obtained on cotton, silk, and wool, which clearly demonstrate that satisfactory leveling properties of the synthesized disperse reactive dye were achieved on all employed substrates in supercritical carbon dioxide.

**Table 3:** Color characteristics of the synthesized anthraquinone dye on different substrates

Fabric	L*	a*	b*	C*	H*	$\bar{K/S}$ [λ_max,_ 505.0 nm]	*σ_K_*_/_ *_S_*
Cotton	81.5	18.7	2.5	18.9	7.5	0.293	0.016
Silk	69.3	29.9	7.5	30.8	14.1	1.108	0.066
Wool	66.4	30.7	12.8	33.2	22.6	1.373	0.041

**Figure 6:**
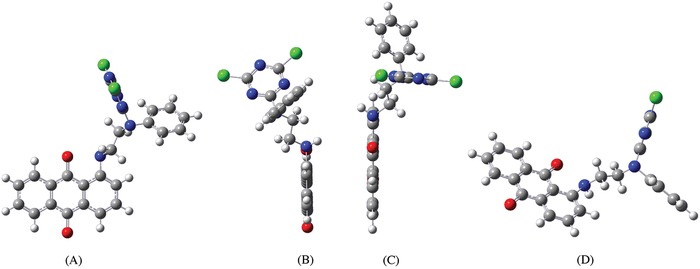
The different conformation views of the disperse reactive dye molecule obtained using the software Gauss View 5.0.8: A) view from *Z*‐axis to *Y*‐axis, B) view from *Y*‐axis to *X*‐axis, C) view from *Y*‐axis to *X*‐axis with a rotation to show the full length of the molecule, D) view from *Y*‐axis to *X*‐axis with a rotation to show the reactive and phenyl groups of the molecule.

Theoretically, as shown in Scheme 1 and as previously demonstrated, the disperse reactive dye was designed with a substituted N‐phenylethylenediamine as a versatile bridge group, which involves hydrophobic phenyl and ethylene groups in its chemical structure and can significantly improve the dye solubility in supercritical carbon dioxide. Therefore, this specially designed versatile bridge group is not only able to play a role in connecting the dye chromophoric matrix and the reactive group (s) to balance the total properties of the dye molecule in structure, but is also readily able to improve the uptake, adsorption, or build up behavior of the dye onto the substrate in hydrophobic media. Moreover, as depicted in **Figure** **6**A–C, a relatively good coplanarity of the dye molecule was also achieved for the designed chemical structure by viewing from different viewing angles, which is helpful for dye diffusion or penetration in the networks of the fiber macrochains due to relatively low steric hindrance. In particular, the utilization of the soft block of the substituted ethylenediamine in the versatile bridge group is also benefit to reduce steric hindrance via readily changing its conformation during dye penetration. Therefore, the designed disperse reactive dye with a versatile bridge group could readily achieve good coloration properties and the desired shades on substrates in a hydrophobic supercritical medium.

#### 2.4.2 Fixation Efficiency of the Uptaken Dye on Substrates

**Figure 7:**
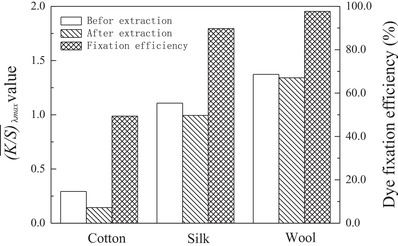
The fixation efficiencies of the anthraquinone disperse reactive dye on cotton, silk, and wool substrates with a dye dosage of 1.0% (o.m.f.) with 0.5% (v/v) acetone in supercritical carbon dioxide under the conditions of 20.0 MPa, 120 °C, and 90 min with a time ratio of fluid circulation to static treatment of 0.10.

A Soxhlet extraction method^3^, ^5^ was utilized for the removal of unfixed dye molecules from cotton, silk, and wool fabrics after a coloration process in supercritical carbon dioxide, as described above. Then, the fixation efficiencies of the uptaken dye on cotton, silk, and wool substrates were obtained, as depicted in **Figure** **7**.

Figure 7 shows that satisfactory and relatively high color intensities on the dyed wool and silk substrates after extraction were observed in comparison with the corresponding values before extraction treatment, accompanied by high fixation efficiencies of the uptaken dye molecules of ≈97.69% and 89.76% for wool and silk, respectively. However, a low color intensity after extraction was also obtained on the cotton sample, resulting in the lowest fixation efficiency of ≈49.37% among all dyed substrates, as shown in Figure 7.

**Scheme 3:**
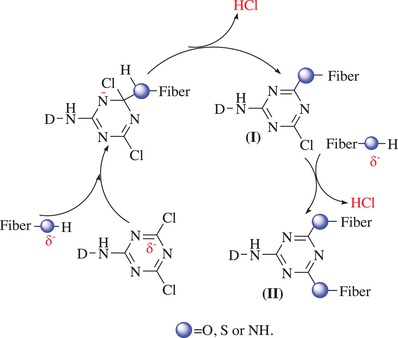
The fixation reaction schematic route and mechanism between the synthesized disperse reactive dye and the functional groups of the substrates in supercritical carbon dioxide.

Theoretically, there is a nucleophilic substitution reaction between the dichlorotriazine reactive groups of the uptaken dye molecules and the functional groups from the macrochains of the substrate fibers; this reaction is responsible for the fixation or bonding of the dye molecules onto the substrate during the dyeing process in supercritical carbon dioxide, as depicted in **Scheme** **3**. Consequently, the ability of the nucleophilic attack of functional groups on the macrochains of different substrates usually affects or even determines the fixation efficiency of the uptaken dye molecules. As well known, amino (—NH_2_), imino (—NH—), and sulfhydryl (—SH) groups in protein‐based fibers, such as wool and silk, are more powerful during nucleophilic substitution in a neutral or acidic medium than hydroxyl groups (—OH) in cellulosic fibers. Therefore, under the same coloration conditions, the uptaken dye molecules more readily formed covalent bonds, such as analogous amido bonds, on the protein‐based fibers than on the cellulosic fibers to form analogous ester bonds in supercritical carbon dioxide. Thus, all these factors resulted in relatively higher dye fixation efficiencies and color intensities on the wool and silk substrates than on the cotton.

#### 2.4.3 Color Fastness Performance

The dyed cotton, silk, and wool samples were further subjected to standard washing and rubbing conditions to evaluate the color fastness performances of the synthesized disperse reactive dye, according to the employed China textile criteria.^29^, ^30^ The achieved results are shown in **Table** **4**.

**Table 4:** Color fastness of the synthesized anthraquinone dye on different substrates

Fabric	Washing fastness							Rubbing fastness	
	Fading	Staining						Dry	Wet
		Cotton	Wool	Acrylic	Polyester	Polyamide‐66	Acetate		
Cotton	3	4–5	4–5	4–5	4–5	4–5	4–5	4–5	3–4
Silk	4	4–5	4–5	5	4–5	4–5	4	4	4
Wool	4–5	4–5	4–5	4–5	4–5	4–5	4–5	4	3–4

Table 4 shows that a good, commercially acceptable color fastness under standard washing conditions was achieved with grades of 4 or 4–5 for the dyed wool and silk substrates, respectively, especially for their staining fastness on the six‐fiber (cotton, wool, acrylic, polyester, polyamide‐66, and acetate) standard adjacent fabric SDC Multifibre DW. Moreover, a satisfactory rubbing fastness of 3–4 and higher grades was also obtained under dry or wet conditions for the dyed protein substrates. Moreover, comparable staining and rubbing fastness at grades 4–5 or 3–4 was also observed between the dyed cotton samples and the dyed protein substrates. However, a lower fading fastness of grade 3 was encountered on the cotton samples than on the protein‐based samples. This is also consistent with the low fixing efficiency of the uptaken disperse reactive dye on cotton fabric, as well as the lower stability of the formed analogous ester bonds between the dye and cotton fibers than that of the analogous amido bonds on the protein fibers when subjected to wet‐chemical conditions for color fastness assessment.

## 3 Conclusions

A novel red‐hued disperse reactive dye involving an anthraquinonoid chromophoric matrix and a versatile bridge group was successfully designed and synthesized by employing a metallic copper‐mediated Ullmann cross‐coupling reaction in a ligand‐free system, followed by a condensation reaction to introduce a dichlorotriazine reactive group. The synthesis parameters by employing the reactants 1‐chloroanthraquinone and N‐phenylethylenediamine were investigated and optimized. Notable influences of the dosages of the solvent (DMF) and the applied KOH, as well as the system temperature and reaction duration, on the isolated yield of the dye precursor were observed. The recommended process for synthesizing the designed novel dye precursor was as follows: react 2.0 mmol of 1‐chloroanthraquinone and 2.0 mmol of N‐phenylethylenediamine with 4.0 mmol of KOH in the presence of 0.1 mmol Cu and 10.0 mL DMF by stirring at 90 °C for 10.0 h. Furthermore, the chemical structure and color characteristics of the synthesized dye were well characterized and verified by FTIR, ^1^H NMR and ^13^C NMR, elemental analysis, LC‐MS, and UV–vis absorption spectroscopy. The preliminary applications in supercritical carbon dioxide further reveal that satisfactory uptake performance, color characteristics, leveling properties, fixation efficiency, and conventional color fastness were successfully achieved on both wool and silk substrates, followed by cotton fabric, by utilizing the synthesized anthraquinonoid disperse reactive dye. All the achieved results clearly prove that the novel disperse reactive dye was well designed and successfully synthesized and is also potentially useful and applicable in the coloration of natural substrates in the textile industry, particularly when employing supercritical carbon dioxide as a medium for sustainable and cleaner production.

## 4 Experimental Section

*Materials and Chemicals*: 1‐Chloroanthraquinone, N‐phenylethylenediamine, copper, and cyanuric chloride were purchased from Aladdin Industrial Corporation (Shanghai, China) in analytically pure grade. KOH in analytically pure grade was provided by Sinopharm Chemical Reagent Co., Ltd. (Shanghai, China). Other utilized reagents, such as DMF, dichloromethane, acetone, and *n*‐hexane, were also analytically pure grade and used without further purification. In addition, silica gel plates (GF254) were provided by Rushan Sanpont New Materials Co. Ltd. (Shanghai, China). Nitrogen gas (N_2_) with a purity higher than (99.99 vol%) and pure carbon dioxide gas (99.6 vol%) were purchased from Suzhou Jinhong Gas Co. Ltd. (Suzhou, China).

Completely pretreated and purified substrates including cotton fabric (100% cotton, plain woven, with a fabric weight of 140.0 g m^−2^), silk fabric (100% silk, plain woven, with a fabric weight of 65.0 g m^−2^), and a worsted fabric (a 2/2↗ twill fabric of 100% wool, with a fabric weight of 200.0 g m^−2^) were used for coloration in supercritical carbon dioxide by employing the obtained disperse reactive dye.

*Synthesizing the Anthraquinonoid Dye Precursor with a 2‐(N‐phenyl)‐Ethylenediamino Bridge Group*: The designed disperse reactive dye precursor of 1‐(2‐(N‐phenyl)‐ethylenediamino)‐9,10‐anthraquinone with an anthraquinonoid chromophoric matrix and a 2‐(N‐phenyl)‐ethylenediamino bridge group was synthesized by employing 1‐chloroanthraquinone and N‐phenylethylenediamine as reactants on the basis of the Ullmann reaction with metallic copper (Cu) as the catalyst in a ligand‐free system. The designed and utilized synthetic route is shown in Step 1 in Scheme 1.

A predetermined 2.00 mmol (0.485 g) of 1‐chloroanthraquinone, 2.00 mmol (260.0 µL) of N‐phenylethylenediamine, 4.00 mmol (0.224 g) of KOH, and 0.10 mmol (0.0060 g) of Cu (copper) were loaded into a 100.0 mL three‐neck flask, and then 10.0 mL DMF was dropwise added into the flask. The synthesizing reaction was carried out under the protection of nitrogen gas for 12.0 h at 100.0 °C in an anhydrous mixed system with continuous stirring. Moreover, the reaction was traced by utilizing thin layer chromatography (TLC) analysis on silica gel plates (GF254, Rushan Sanpont New Materials Co., Ltd, Shanghai, China), and the retardation factor (*R*_f_) value for the target product of the designed dye precursor was 0.30 (petroleum ether and dichloromethane, 1:2, v/v).

Afterward, once the reaction reached completion, the product mixture in the flask was cooled to ambient temperature and then filtered and washed fully with dichloromethane. The achieved filtrate was further extracted with dichloromethane, accompanied by full washing of the organic layer with deionized water. Then, the extracted organic layer of dichloromethane solution containing the dye precursor was concentrated and subsequently treated by vacuum‐drying to obtain a reddish brown solid powder of a crude product of the dye precursor. The crude product was further purified by silica gel column chromatography by employing petroleum ether and dichloromethane (3:2, v/v) as the eluent. Finally, a solid dark red powder of the desired dye precursor was collected at an isolated yield of 60.88%.

*Bonding a Reactive Group onto the Anthraquinonoid Dye Precursor via the Bridge Group*: The designed final product of the anthraquinonoid disperse reactive dye was further synthesized by bonding the reactive group of cyanuric chloride onto the previously achieved dye precursor via the bridge group of 2‐(N‐phenyl)‐ethylenediamino on the basis of a condensation reaction according to the proposed reaction route of step 2 in Scheme 1.

A quantitative amount of 5.0 mL 1,4‐dioxane was used to dissolve a predetermined 1.50 mmol (0.277 g) of cyanuric chloride, and then the solution was cooled in a low‐temperature bath of anhydrous ethanol at a temperature range of 0–5 °C. Then, 1.0 mmol (0.342 g) of the anthraquinonoid dye precursor was dissolved in 15.0 mL 1,4‐dioxane solution, and 1.0 mmol (0.106 g) of Na_2_CO_3_ was dissolved in 15.0 mL water. Afterward, both solutions were dropwise added into the reaction system at the same time. Therefore, the condensation reaction to bond the reactive group onto the dye precursor was implemented for 3.0 h at a temperature of 0.0–5.0 °C with stirring. TLC analysis was also carried out to trace the progress of the condensation reaction, and the *R*_f_ value for the target product was 0.22 (petroleum ether and dichloromethane, 1:2, v/v). After the reaction was completed, the obtained mixture was diluted with deionized water to ≈350 mL to precipitate the target product. Then, a crude product in the form of a red solid mainly containing the anthraquinonoid disperse reactive dye bonded with a reactive group of cyanuric chloride was filtered, washed, and dried under vacuum at room temperature. Additionally, the achieved crude dye product was further purified by employing silica gel column chromatography with petroleum ether and dichloromethane (1:1, v/v) as the eluent. Finally, a bright red solid powder of the designed and synthesized disperse reactive dye was obtained with an isolated yield of 60%.

*Characterization and Analysis of the Synthesized Disperse Reactive Dye and Its Precursor*: Elemental analysis (EA) of the purified final dye product was performed on an Elementar CHN analyzer (Vario EL III, Elementar Analysensysteme GmbH, Hanau, Germany) to determine the elemental components of carbon, hydrogen, and nitrogen in the chemical structure. Moreover, LC‐MS spectra were recorded by employing a quadrupole/time‐of‐flight tandem mass spectrometer (micrOTOF‐Q III, Bruker Daltonics Inc., USA) with a flow rate of dry gas at 4.0 L min^−1^ and a pressure of 0.5 bar for the nebulizer in a positive ion mode. ^1^H NMR spectra for the purified disperse reactive dye as well as its precursor were recorded by employing a Bruker Avance III 400 Hz NMR spectrometer (400 MHz for ^1^H NMR; Bruker BioSpin GmbH, Rheinstetten, Germany) with a solvent of deuterated chloroform (CDCl_3_), and tetramethylsilane was employed as an internal standard. Furthermore, ^13^C NMR spectra of the achieved dye were measured on a Agilent 600 MHz DD2 NMR (151 MHz for ^1^C NMR; Agilent Technologies Corporation, USA) under the same solvent and internal standard conditions as used for the ^1^H NMR spectra. The FT‐IR spectra of the synthesized dye and its precursor were also detected with the potassium bromide wafer method in a region of 400.0–4000.0 cm^−1^ on a Nicolet 5700 spectrometer (Thermo Nicolet Corporation, Madison, WI, USA).

UV–vis absorption spectra of the obtained dye precursor and its final product were further investigated on a UV–vis spectrophotometer (TU‐1810, Beijing Purkinje General Co., Ltd, Beijing, China) with concentrations ranging from 7.56 × 10^−5^ mol L^−1^ to 9.66 × 10^−5^ mol L^−1^ in different media, such as dichloromethane, diluted acidic and alkaline solutions, DMSO, DMF, ethanol, and *n*‐hexane. Additionally, the melting point (M. P.) of the synthesized final product was measured on a digital melting‐point apparatus (WRR, Shanghai Precision and Scientific Instruments, Shanghai, China) in degrees Celsius (°C) with a heating rate of 1.0 °C min^−1^.

*The Obtained Characteristic Data for the Chemical Structure and Properties of the Dye Precursor and Its Final Product*: The characteristic data for the achieved and purified dye precursor are listed as follows. *R*_f_ (Retardation factor) = 0.30 (petroleum ether and dichloromethane, 1:2, v/v). FT‐IR (KBr) υ cm^−1^: 3372, 3248, 1632, 1604 (N—H); 3049, 3021 (=C—H); 2929, 2870, 1460 (—CH_2_—); 1654 (C=O). ^1^H NMR (400 MHz, CDC1_3_) δ ppm: 9.89 (s, 1H, H‐8′), 8.26 (dd, *J* = 10.1, 8.1 Hz, 2H, H‐1′, H‐4′), 7.74 (dt, *J* = 14.8, 6.8 Hz, 2H, H‐2′, H‐3′), 7.62 (d, *J* = 7.0 Hz, 1H, H‐5′), 7.54 (t, *J* = 7.9 Hz, 1H, H‐6′), 7.21 (t, *J* = 7.8 Hz, 2H, H‐13′, H‐15′), 7.08 (d, *J* = 8.5 Hz, 1H, H‐7′), 6.75 (t, *J* = 7.3 Hz, 1H, H‐14′), 6.69 (d, *J* = 7.9 Hz, 2H, H‐12′, H‐16′), 3.98 (s, 1H, H‐11′), 3.63 (dd, *J* = 11.3, 5.6 Hz, 2H, H‐9′), 3.55 (d, *J* = 5.5 Hz, 2H, H‐10′).

The characteristic data for the final dye product are also listed as follows. *R*_f_ = 0.22 (petroleum ether and dichloromethane, 1:2, v/v). FT‐IR (KBr) υ cm^−1^: 3261, 1630 (N—H); 3070 (=C—H); 2928, 2853, 1451 (—CH_2_—); 1661 (C=O); 1552 (C=N); 796 (C—Cl).

^1^H NMR (400 MHz, CDCl_3_) δ ppm: 9.84 (t, *J* = 5.4 Hz, 1H, H‐8), 8.24 (d, *J* = 8.1 Hz, 2H, H‐1, H‐4), 7.74 (dt, *J* = 15.0, 7.0 Hz, 2H, H‐2, H‐3), 7.65 (d, *J* = 7.0 Hz, 1H, H‐5), 7.59 (t, *J* = 7.9 Hz, 1H, H‐6), 7.47 (t, *J* = 7.6 Hz, 2H, H‐13, H‐15), 7.43‐7.32 (m, 2H, H‐7, H‐14), 7.24 (d, *J* = 7.6 Hz, 2H, H‐12, H‐16), 4.30 (t, *J* = 7.0 Hz, 2H, H‐10), 3.66 (dd, *J* = 13.3, 6.4 Hz, 2H, H‐9).

^13^C NMR (151 MHz, CDCl_3_) δ ppm: 185.26 (s, C‐14), 183.65 (s, C‐7), 170.65 (s, C‐23), 170.46 (s, C‐24, C‐25), 165.71 (s, C‐12), 151.31 (s, C‐17), 140.28 (s, C‐10), 135.52 (s, C‐8), 134.78 (s, C‐6), 134.03 (s, C‐4), 133.15 (s, C‐3), 132.97 (s, C‐1), 129.91 (s, C‐19, C‐21), 128.39 (s, C‐11), 126.97 (s, C‐20), 126.77 (d, *J* = 8.7 Hz, C‐2, C‐5), 117.81 (s, C‐9), 116.37 (s, C‐18, C‐22), 113.66 (s, C‐13), 49.69 (s, C‐16), 39.75 (s, C‐15).

The EA (%) for C_25_H_17_Cl_2_N_5_O_2_ is listed as follows: the calculated values for different components were C 61.24, H 3.49, and N 14.28; the experimentally found values were C 61.11, H 4.16, and N 13.49. The LC‐MS analysis data are as follows: the theoretical value was *m*/*z* = 489.1, and the experimental values were [M + H]^+^ = 490.2, [M + 3]^+^ = [M + H + 2]^+^ = 492.1, [M + 5]^+^ = [M + H + 4]^+^ = 494.2.

*Dyeing of Cotton, Silk, and Wool Substrates with the Obtained Disperse Reactive Dye in SCF‐CO_2_ Medium*: The application of the designed and synthesized disperse reactive dye involving the versatile bridge group and the reactive group of dichloro‐S‐triazine was carried out to dye natural substrates of cotton, silk, and wool in supercritical carbon dioxide by employing a self‐built system constructed by our research group as described elsewhere.^10^, ^28^

The natural fabric samples of cotton, wool, or silk to be dyed were each wrapped around an improved dyeing beam, as shown in our previous work,^28^ and then the beam was set in the dyeing vessel in each dyeing experiment. An aliquot of 1.0% o.m.f. (on the mass of fabric) of the obtained dye powder with 0.5% (v/v) acetone to improve solubility was also charged into the dyeing vessel. Thereafter, the dyeing vessel was sealed, and the whole system was ready for pressurizing and heating of the CO_2_ medium. When the pressure and temperature of the dyeing system were attained under the designated conditions, the supercritical dyeing process was carried out at 20.0 MPa and 120 °C by circulating the supercritical carbon dioxide fluid containing the dissolved dye and solvent with a syringe pump at a designated time ratio (*R*_time_) of 0.10 of fluid circulation relative to the static dyeing duration. Consequently, the dissolved disperse reactive dye molecules from the dyeing media were taken up onto the surfaces of the natural fibers with subsequent further penetration into the amorphous fiber regions. Crucially, the uptake dye molecules could also be fixed on the substrates during the dyeing process via a series of reactions between the reactive groups on the dye molecules and the functional groups on the macrochains of the substrate fibers, such as the amino groups on wool and silk, hydroxyl groups on cotton, etc. After coloration for 90.0 min, the dyeing process was terminated by a subsequent cleaning procedure with fresh supercritical carbon dioxide at 20.0 MPa and 80 °C for 20.0 min, and then the dyeing system was depressurized for the recovery of the CO_2_ gas. Finally, the colored substrate sample was removed from the dyeing vessel for measurements without any further treatment. Moreover, the whole dyeing system was fully cleaned by fresh supercritical carbon dioxide fluid after every run; similar procedures and operations were referenced in our previous report.^10^, ^28^

*Measurements of the Color Characteristics and Properties on Natural Fiber Substrates*: The color characteristics, including the colorimetric parameters (L*, a*, b*, C*, H*) and the color intensity (*K*/*S* values), for the obtained disperse reactive dye on cotton, silk, and wool substrates were measured on a HunterLab UltraScan PRO reflectance spectrophotometer (HunterLab. Co., Ltd., Reston, USA) by employing a simulated D_65_ light source lamp and a 10° visual angle. A fourfold form for wool and cotton samples and an eightfold form for the silk substrate were utilized during the color measurements. The conventional Kubelka–Munk equation was also utilized to calculate the *K*/*S* and $\bar{K/S}$ values of the dyed fabric samples at a maximum absorption wavenumber (λ_max_) of 505.0 nm for the synthesized disperse reactive dye, as described elsewhere.^10^, ^28^ Moreover, the leveling properties of the dye on the different fabric substrates were assessed according to a method similar to that in our previous work by employing the standard deviation (σ_K/S_) of the *K*/*S* values,^10^, ^28^ and the dye fixation efficiency on the substrates was determined according to the literature.^10^, ^28^

The washing fastness of the dyed natural cotton, silk, and wool fabric substrates was tested on a washing fastness apparatus (SW‐12A; Changzhou Depu Textile Technology Co., Ltd, China) with a liquor ratio of 1:50 and 5.0 g L^−1^ soap powder at 40.0 °C for 30.0 min according to the Chinese textile criteria GB/T 3921.1‐2008A (1) (which is equivalent to ISO 105‐C10:2006A(1)),^29^ and an adjacent fabric of SDC Multifibre DW (product code 2115; SDC Enterprises Co., Ltd, Bradford, UK) was used for staining fastness assessment. The rubbing fastness evaluation for the dyed fabric substrates was carried out on a rubbing tester (Y571B; Nantong Hongda Experiment Instruments Co., Ltd, Nantong, China) with dry and wet samples according to GB/T 3920‐2008 (which is equivalent to ISO 105‐X12:2001).^30^

*Caution*: The coloration of substrates in supercritical carbon dioxide in this work involved a high‐pressure equipment system, which was operated by professional operators.

## Conflict of Interest

The authors declare no conflict of interest.

## Supporting information

Supplementary
