# Supplementary material for: Synthesis of a Novel Disperse Reactive Dye Involving a Versatile Bridge Group for the Sustainable Coloration of Natural Fibers in Supercritical Carbon Dioxide
Source: Adv Sci (Weinh). 2018 Nov 8;6(1):1801368. doi: 10.1002/advs.201801368 (PMC6325576; doi:10.1002/advs.201801368)
Supplement: Supplementary file 1 — Supplementary [file ADVS-6-1801368-s001.pdf]

Copyright WILEY-VCH Verlag GmbH & Co. KGaA, 69469 Weinheim, Germany, 2018.

ADVANCED SCIENCE
Open Access

Supporting Information

for *Adv. Sci.,* DOI: 10.1002/advs.201801368

Synthesis of a Novel Disperse Reactive Dye Involving a Versatile Bridge Group for the Sustainable Coloration of Natural Fibers in Supercritical Carbon Dioxide

*Yue Fan, Yan-Qin Zhang, Kai Yan, and Jia-Jie Long**

Copyright WILEY-VCH Verlag GmbH & Co. KGaA, 69469 Weinheim, Germany, 2016.

Supporting Information

Synthesis of a novel disperse reactive dye involving a versatile bridge group for the sustainable coloration of natural fibers in supercritical carbon dioxide

Yue Fan[1], Yan-Qin Zhang[1], Kai Yan[1], Jia-Jie Long[1]*

[1] College of Textile and Clothing Engineering, Soochow University, Suzhou 215123, China;

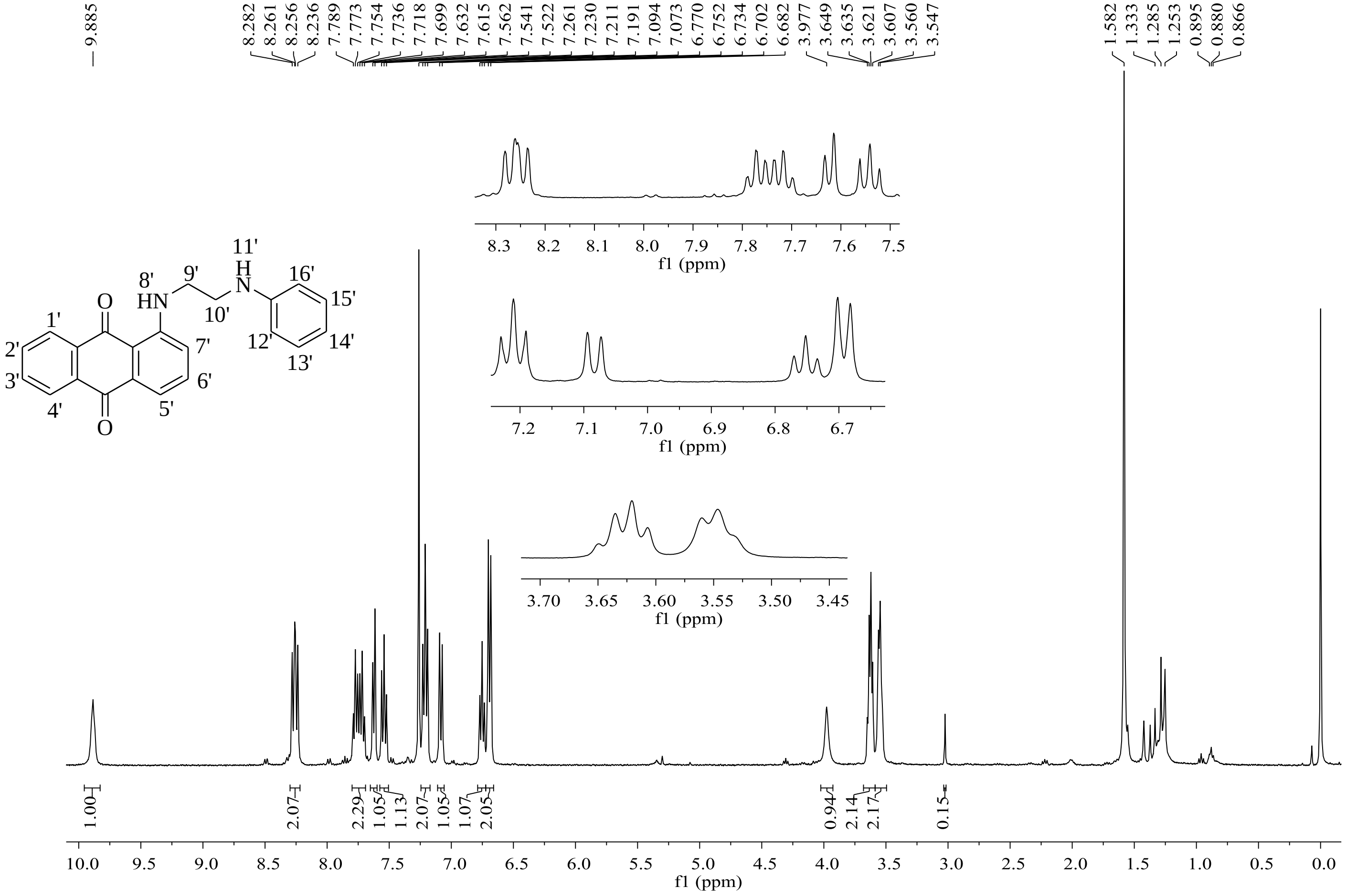

Figure CP 1 ^{1}H NMR spectra of the dye precursor

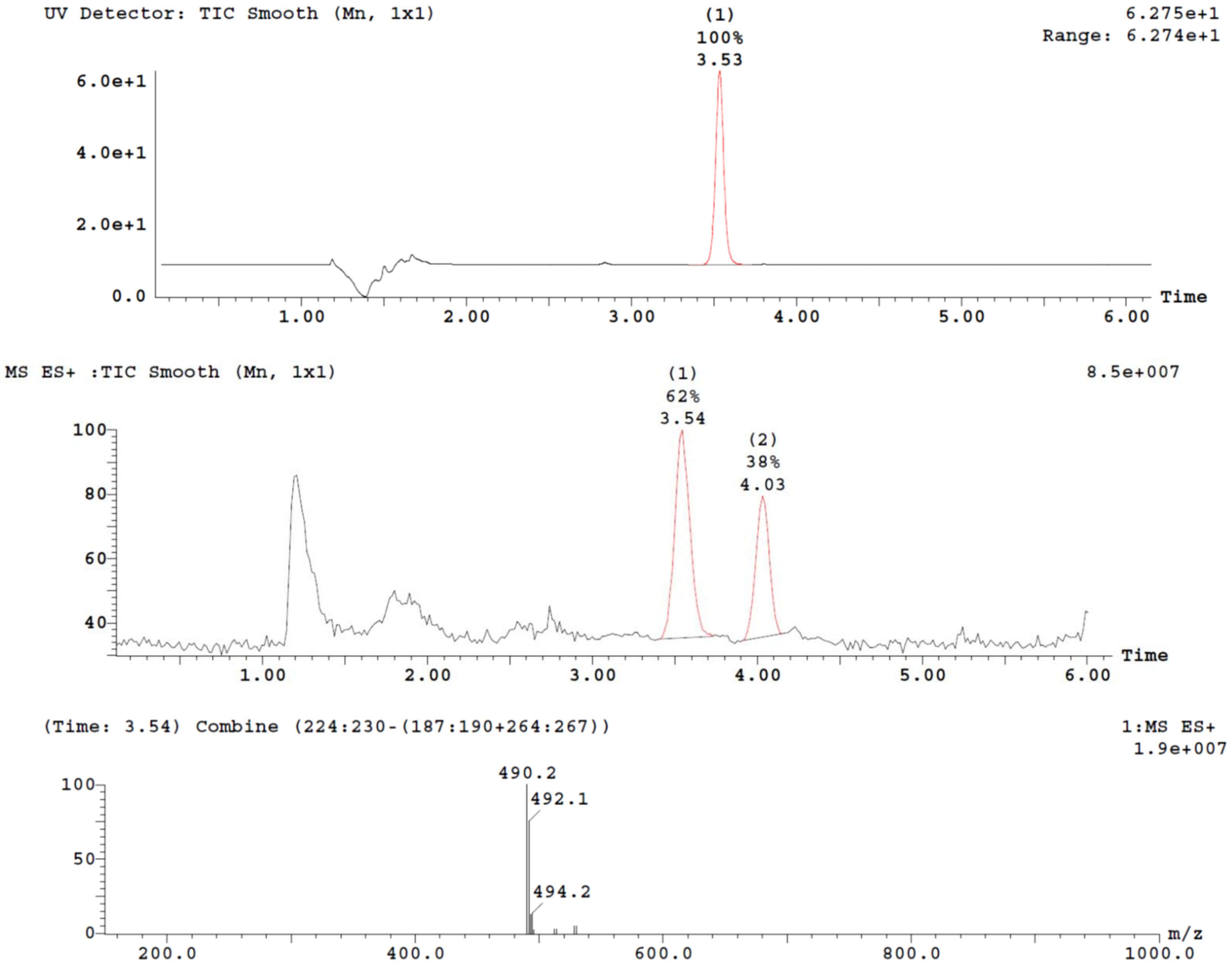

Figure CP 2 **LC-MS analysis of the anthraquinone disperse reactive dye**
